# Notes and News

**Published:** 1891-03-07

**Authors:** 


### Notes and News.

Collected and Communicated.

The burden of what was said at the annual meeting of the General Council of the Great Northern Central Hospital on the 27th ult., was the urgent need for the completion of the buildings, the indoor accommodation being found quite insufficient to meet the demands made upon it. In consequence of this a great many serious cases seeking admission had been sent to other hospitals, or had been obliged to wait a long time before they could be relieved. Mr. C. T. Murdoch, M.P., who presided, trusted that renewed efforts would be made to secure funds to carry out this very important work. £20,000, it is estimated, is needed, of which the hospital has but £1,000. Despite the fact that 131 more in-patients had been treated than in the previous year, the expenditure was less than in 1889.

It is something, in these sensational days, to hear of a work of women for women, which has been steadily carried on for thirty-four years without advertising itself or appealing to the public in any way. Such is the record of the Bible-women and District Nurses' Mission, which held its first public meeting on February 27th, in the Prince's Hall. The meeting was large and sympathetic, amply proving, if proof were needed, that a good work can make itself friends without the aid of speeches and circulars. The Earl of Harrowby presided, and gave an interesting sketch of the growth and work of the mission since it was founded by Mrs. Ranyard. It would be difficult to gauge the quiet influence for good exercised by these workers, moved, as they are, by a spirit of love and self-sacrifice. The Bishop of Winchester gave a stirring address in which he expressed his sympathy with the mission and his approval of the way in which the work was carried on. Sir Dyce Duckworth, M.D., bore testimony to the valuable aid which the District Nurses give to the sick poor.

The festival dinner at the Hotel Metropole, on Monday, in aid of the East London Hospital for Children, was a very bright and well-managed function. The Duke of Portland was an enthusiastic chairman, speaking with an evident knowledge of his subject, and speaking clearly and distinctly so as to be heard all over the hall. He remarked that the committee of the East London Hospital was no mere ornamental figure-head, but a real hard-working body, and then went on to mention with eulogy Mr. Cheston's name, which was received with loud applause. Mr. Cheston made an amusing and interesting speech, and referring to General Booth's scheme begged those present not to forsake old and tried charities for new schemes. The East London Hospital sadly needed an enlarged out-patient department and a home for the nurses; these necessary buildings would cost £7,000, and the hospital hoped to begin them next March, but nothing would induce it to run into debt. Subscriptions amounting to £4,520 were announced as the result of the dinner, the Duke of Portland having given £200. Lord Kilmarnock, Dr. Dawson Williams, Mr. Charrington, M.P., and others spoke, and there was some delightful music between the speeches. Everyone present must have been much impressed by the necessity of helping the East London Hospital, and must have recognised, from what they heard, that the charity is one of the most deserving in this great city of ours.

It is satisfactory to notice that the relief afforded by the Cancer Hospital (Free) Brompton, continues to increase year by year. The report for 1890, which was read at the annual general meeting of Governors, shows that last year 1,789 new patients were received, of whom 630 were in-patients, the total number of out-patients being 6,639. This increase in the work points to the necessity for increased funds, the reliable income being about £3,000 a-year less than the ordinary expenditure. The Samaritan Fund has proved very useful for assisting discharged patients.

The fifty-second annual meeting of the supporters of the King's College Hospital was held on February 25th, Mr. Richard Twining occupying the chair. The report showed that the hospital is now maintaining on the average more than 200 beds. The total number of in-patients in 1890 was 2,518, while the number of attendances in the out-patient department was 29,556. Although these numbers show a considerable increase on those of 1889, the total increase of expenditure during 1890 was only £733 11s., and in return for this the 15 beds in the Todd Ward have been open during the whole year, and those in the Cheere Ward during the last two months. The report was unanimously agreed to, and the rest of the business, which was of a formal character, having been gone through, the proceedings terminated.

"Looking a gift horse in the mouth" is not a gracious proceeding under any circumstances, but many people will be surprised at the length to which it is being carried by the authorities of the Manchester Royal Infirmary in the matter of the handsome offer by the Whitworth Trustees, of a most excellent site for a new hospital to be worked in connection with both the Royal Infirmary and the medical school of Owens College. The inadequacy of the Infirmary to meet the ever-increasing requirements of the sick poor is acknowledged, and at the same time just complaints are made of the absence of covenient facilities for clinical instruction for the students of Owens College. These wants would all be met by the establishment of the proposed College Hospital at Stanley Grove, in close proximity to the college, yet not too far from the most densely-populated parts of the city. From the point of view of the public interest it is not easy to appreciate the reasons which the Infirmary authorities doubtless have for raising difficulties in the way of a satisfactory arrangement. We hope that the joint committee which has been appointed at the instance of Principal Ward, will be successful in finding a way of reconciling the conflicting claims and interests.

The Secretary-Superintendent of the Middlesex Hospital (Mr. F. Clare Melhado), at a quarterly meeting held on the 26th ult., under the presidency of Mr. R. Ruthven Pym, Senior Treasurer, reported that the capital fund of the hospital had been augmented during the year 1890 to the extent of £83,433, of which the great bulk (some £77,800) had been left to this charity by one generous donor, the late Miss Mary Eason. The accounts for 1890, when compared with previous years, were fairly satisfactory, for the income (£20,635) showed an increase of £859, while the expenditure, both by reason of there being no special undertaking (such as the erection of the new post-mortem room and mortuary last year) and by reason of sundry minor economies, had, in spite of the increased number of patients and staff, been reduced by £1,473 (from £28,590 to £27,117). Even this reduction has failed, however, to bring the income within £6,000 of the expenditure, so that this latter sum has had to be taken out of the capital. During the past year 3,109 in-patients (161 more than in 1889) had been treated, making the average number of beds occupied daily 250. The out-patients numbered 38,800, and the attendances they recorded were 94,346. In conclusion, it may be mentioned that the grant received by the Middlesex from the Hospital Sunday Fund was over £50 larger than last year's, is the highest the hospital has ever earned, and is the second biggest grant ever awarded by the Fund to any hospital.

On the 25th ult. the Hospitals Association held its fourth general meeting of the present session at Westminster Hospital, when Dr. Richard Greene, of Northampton, read a paper on "Hospitals for the Insane, and Clinical Instruction in Asylums." The chair was taken at eight o'clock by Chas. A. Lockhart Robertson, Esq., M.D., F.R.C.P., a Lord Chancellor's Visitor in Lunacy, and among those present were Mr. G. F. Blandford, Mr. D. Bower, Mr. Henry C. Burdett, Mr. W. Harding, Mr. Lewis Haslam, Mr. P. Mitchelli, Mr. G. H. Savage, Mr. Seymour Tuke, Mr. C. E. Saunders, Mr. S. W. D. Williams, the Rev. James Mahomed, and Surgeon-Major Macdonnell.

In the discussion which followed, Dr. Tuke expressed doubts as to the cause of the cure for insanity being advanced by recent proposals on the subject, and held that on the whole the existing asylums were at present the great and best means for curing those mentally afflicted, whether rich or poor. At the same time he appreciated, and perhaps more fully than many, the value of properly conducted lunatic wards in workhouses for a certain class of chronic cases. As to the question of clinical instruction in asylums, he considered it a most important one, and regretted the general absence of scientific enthusiasm, more especially when the vast amount of pathological material available for research was considered, but he recognised the fact that the amount of work to be done in asylums by those in medical charge was largely the cause of this state of things.

Dr. Blandford, while admitting his ignorance of the details of the proposed London hospital for acute cases, was so far acquainted with the proposal as to ask where in central London a hospital of even only 100 beds could be established? Criticising the treatment suggested in the new institution, Dr. Blandford said a sort of idea had entered the heads of those with whom the scheme had originated, that the patients must be put in wards and kept in bed more or less under medical treatment, with all the resources of modern surgery and medicine turned on to cure them. A good deal he knew could be done by these, but only in conjunction with other adjuncts unobtainable in an asylum in a large city like London.

Mr. Henry C. Burdett thought it most desirable that the public should be guided on this question, for it would be monstrous if the ratepayers' money were wasted for purposes held and believed to be impossible. On the questions of hospitals for the insane, it seemed to him to be of the greatest importance that all having experience should state their opinions fully, and he considered it most desirable that they should have guidance as to what the faculty really thought on the question of a metropolitan hospital for acute cases of insanity.

Dr. Savage was not at all satisfied with county asylums, and expressed himself as favouring a modification of asylums on the hospital system, for it seemed quite impossible to him that 1,000 persons, including, perhaps, 10 or 20 per cent. of acute cases, could be properly treated, from a medical point of view, as in the large asylums. Insanity—which he considered a bad expression—might include a disease of the body expressing itself in a disordered mind. He cordially agreed with Dr. Greene's views.

Dr. Harding, speaking from an assistant medical officer's point of view, expressed the regret and disappointment they had felt at the direction in which the scheme of the proposed hospital had drifted. He fully recognized the importance of clinical instruction in asylums, and thought it ought to be made compulsory.

Dr. Greene having briefly replied to the various points raised, the Chairman, in conclusion, commented upon the discharge of patients apparently cured, but whose liberation was a source of harm to society no less than to themselves; and he expressed his very strong sense of the terrible evils attendant upon ill-considered marriages, the appalling results of which, he was sure, every medical man in that room had familiar and terrible experience in the course of his practice. Votes of thanks to the governors of Westminster Hospital and the chairman concluded the meeting.

